# 1-Methyl-1′-(4-methyl­phen­yl)-2′,3′,5′,6′,7′,7a’-hexa­hydro-1′*H*-dispiro­[piperidine-3,2′-pyrrolizine-3′,3′′-indoline]-4,2′′-dione

**DOI:** 10.1107/S1600536812036240

**Published:** 2012-08-25

**Authors:** S. Ramaswamy, R. Sribala, N. Srinivasan, R. V. Krishnakumar, R. Ranjith Kumar

**Affiliations:** a Department of Physics, N.M.S.S. Vellaichamy Nadar College, Madurai 625 019, India; b Department of Physics, Thiagarajar College, Madurai 625 009, India; c School of Chemistry, Madurai Kamaraj University, Madurai 625 021, India

## Abstract

The title compound, C_26_H_29_N_3_O_2_, crystallizes with two mol­ecules in the asymmetric unit, having C—H⋯O inter­actions between them and resulting in a dimer characterized by an *R*_2_^2^(11) motif. These dimers are linked into an *ABABAB* chain *via* N—H⋯O, N—H⋯N and C—H⋯O built edge-fused *R*_1_^2^(5) and *R*_2_^2^(7) motifs. This chain is linked to its inversion-related partner *via* N—H⋯O bonds with an *R* ^2^_2_(8) motif and leads to a double chain extending along the *b* axis characterized by an *R*_6_^6^(36) motif across the inversion centres. The methyl group of the phenyl ring and the oxindole of mol­ecule *A* and *B* are involved in C—H⋯π inter­actions. One C atom of the pyrrolizine ring of mol­ecule *A* and its attached H atoms show positional disorder, the major and minor components being in the ratio 0.706 (7):0.294 (7).

## Related literature

For ring puckering parameters, see: Cremer & Pople (1975 ▶). For hydrogen-bond motifs, see: Bernstein *et al.* (1995 ▶).
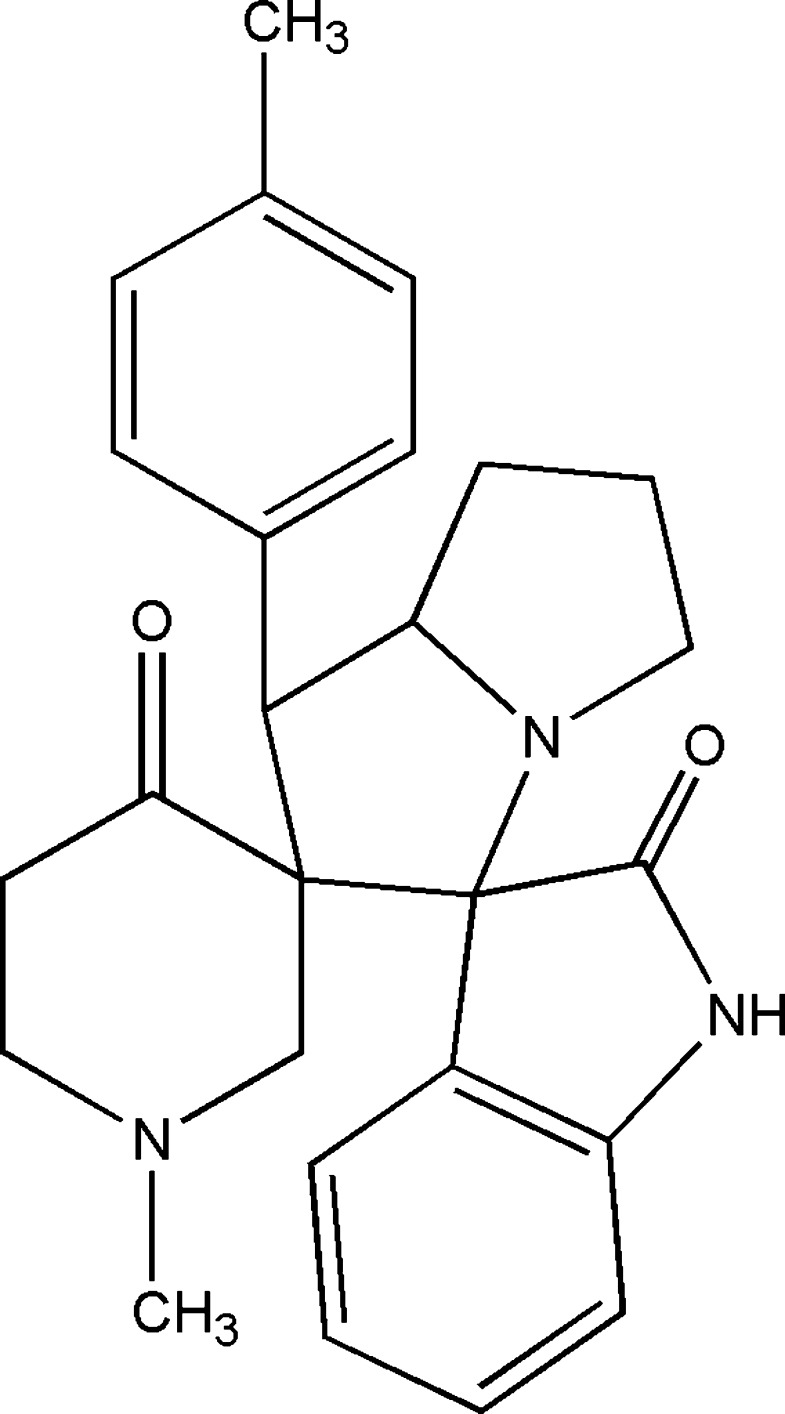


## Experimental

### 

#### Crystal data

C_26_H_29_N_3_O_2_
*M* *_r_* = 415.52
Triclinic, 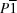

*a* = 8.7516 (4) Å
*b* = 12.4649 (5) Å
*c* = 21.4605 (8) Å
α = 97.654 (2)°
β = 101.024 (2)°
γ = 102.345 (2)°
*V* = 2207.45 (16) Å^3^
*Z* = 4
Mo *K*α radiation
μ = 0.08 mm^−1^
*T* = 293 K
0.28 × 0.19 × 0.19 mm

#### Data collection

Bruker Kappa APEXII CCD diffractometer
Absorption correction: multi-scan (*SADABS*; Bruker, 2009 ▶) *T*_min_ = 0.93, *T*_max_ = 0.96
44464 measured reflections
7765 independent reflections
5493 reflections with *I* > 2σ(*I*)
*R*_int_ = 0.037

#### Refinement

*R*[*F* ^2^ > 2σ(*F* ^2^)] = 0.040
*wR*(*F* ^2^) = 0.110
*S* = 1.02
7765 reflections
574 parameters
H-atom parameters constrained
Δρ_max_ = 0.21 e Å^−3^
Δρ_min_ = −0.17 e Å^−3^

### 

Data collection: *APEX2* (Bruker, 2009 ▶); cell refinement: *SAINT* (Bruker, 2009 ▶); data reduction: *SAINT*; program(s) used to solve structure: *SHELXS97* (Sheldrick, 2008 ▶); program(s) used to refine structure: *SHELXL97* (Sheldrick, 2008 ▶); molecular graphics: *PLUTON* (Spek, 2009 ▶); software used to prepare material for publication: *SHELXL97*.

## Supplementary Material

Crystal structure: contains datablock(s) I, global. DOI: 10.1107/S1600536812036240/fj2583sup1.cif

Structure factors: contains datablock(s) I. DOI: 10.1107/S1600536812036240/fj2583Isup2.hkl

Additional supplementary materials:  crystallographic information; 3D view; checkCIF report

## Figures and Tables

**Table 1:** Hydrogen-bond geometry (Å, °) *Cg*1 and *Cg*2 are the centroids of the C20*A*–C25*A* and C20*B*–C25*B* rings, respectively.

*D*—H⋯*A*	*D*—H	H⋯*A*	*D*⋯*A*	*D*—H⋯*A*
N2*A*—H2*A*⋯O1*A* ^i^	0.86	1.99	2.8331 (17)	167
N2*B*—H2*B*⋯O1*A* ^ii^	0.86	2.48	3.0829 (18)	128
N2*B*—H2*B*⋯N1*A* ^ii^	0.86	2.24	3.0461 (18)	156
C4*A*—H4*A*⋯O1*B* ^iii^	0.98	2.52	3.4598 (19)	160
C16*B*—H16*C*⋯O2*A*	0.97	2.47	3.431 (2)	171
C5*A*—H52*A*⋯O2*B*	0.97	2.58	3.404 (2)	143
C6*A*—H63*A*⋯*Cg*1^iv^	0.97	2.97	3.819 (9)	147
C6*B*—H62*B*⋯*Cg*2^iv^	0.97	2.93	3.827 (3)	155

Symmetry codes: (i) 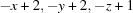; (ii) 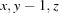; (iii) 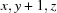; (iv) 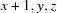.

**Fig. 1.**
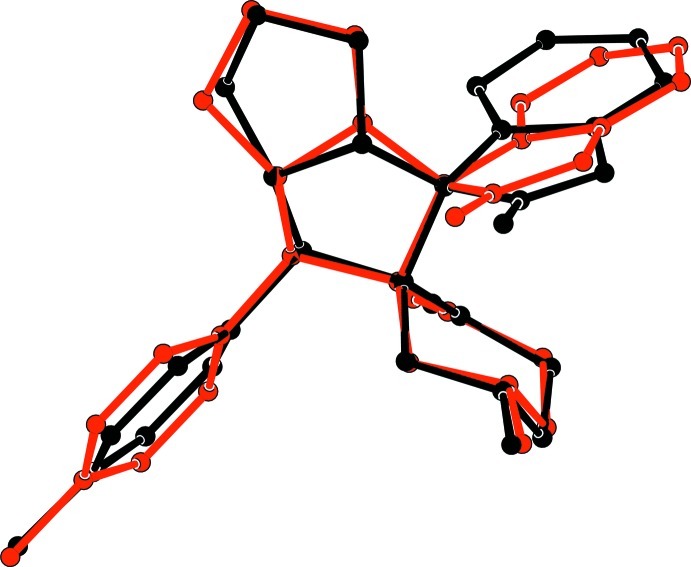
Overlay diagram of the two molecules A (Red) and B (Black) in the asymmetric unit

**Fig. 2.**
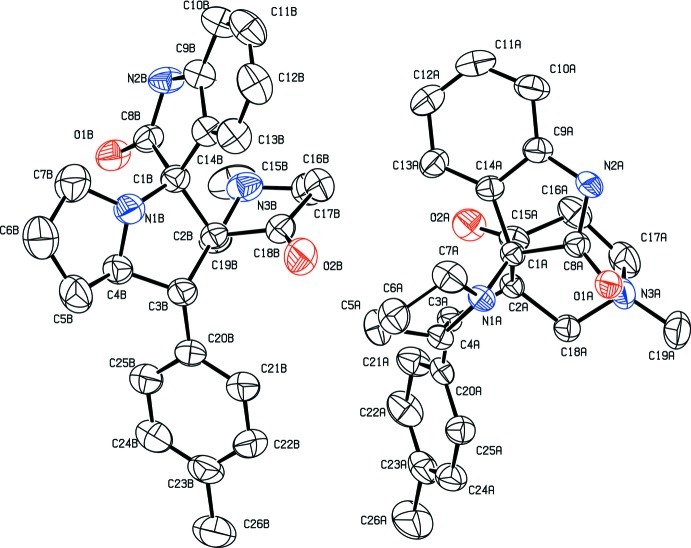
The labelling scheme of molecule A with 50% probability displacement ellipsoids for non-H atoms. H atoms have been omitted for clarity.

**Fig. 3.**
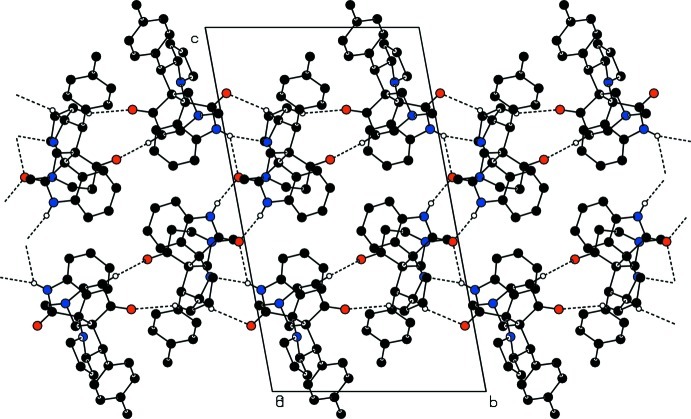
A view of the molecular aggregation along the b--axis. H atoms that are not involved in hydrogen bonding have been omitted for clarity.

## supplementary crystallographic information

### Comment

The title compound contains two molecules in the asymmetric unit forming an asymmetric dimer through C—H···O hydrogen bonds between them. The molecular structure with displacement ellipsoids drawn at 50% probability level is shown in Fig.1. The C8—O1 distance in the indolone in molecules A and B are 1.2244 (18)and 1.215 (2) A°, respectively. The difference in the C8—O1 bond distances in molecules A and B is substantiated by the deviation of O1 from the plane by about 0.4122 (18) in molecule A and 0.2907 (21) in molecule B. This deviation of O1 from planarity seems to have considerably influenced differences in the values of the torsion angles N1—C1–C8—O1 and C2—C1—C8—O1 of molecules A and B. In molecule A, these torsion angles are 37.5 (2) and -75.6 (2)° while in B these values are -53.8 (2) and 61.0 (2)°, respectively. These differences in conformation may also be attributed to a significant intermolecular feature that O1A participates in a three-centered hydrogen bond involving N—H···O and N—H···N types and O1B in a C—H···O type hydrogen bond.

The carbon atom C6 in the hexahydropyrrolizine ring of molecule A shows positional disorder with major and minor component of 0.71 and 0.29, respectively for its site occupancy factor. This disorder leads to a flipping of the conformation of the N1—C4—C5—C6—C7 ring with the puckering parameters (Cremer & Pople, 1975) observed as twisted on C5—C6 with q=0.393 Å, φ=271.4 (3)° for the major component and twisted on C6—C7 with q=0.347 (5) Å, φ=125.0 (7) ° for the minor component. The corresponding ring in molecule B shows similar twist conformation but with the twist on N1—C4 with q=0.394 (2) Å, φ=15.9 (4) °. The puckering of the five-membered ring N1—C1—C2—C3—C4 of the pyrrolizine is envelope (^3^E) on atom C2 with q = 0.3615 (17) Å, φ = 66.3 (3)° in molecule A and envelope (E_5_) on atom C4 with q = 0.4364 (19) Å, φ = 320.7 (2) ° in molecule B. The puckering of the piperidinone rings in both the molecules is close to the usual chair with Q = 0.551 (2) Å, θ = 24.0 (2)°, φ = 353.7 (8)° for molecule A and Q = 0.559 (2) Å, θ = 16.4 (2)°, φ = 339.4 (8)° for molecule B. The hydrogen-bonded interaction (Table 1) between molecules generate one-dimensional double chains extending along the *b* axis (Fig.2).

The molecular interaction pattern is characterized by four different graph-set motifs (Bernstein *et al.*, 1995) *viz.**R*^2^_2_(8), *R*^2^_2_(7), *R*^2^_1_(5) and a *R*^2^_2_(11) type. The *R*^2^_2_(11) motif occurs between the two molecules in the asymmetric unit through C—H···O hydrogen bonds. The *R*^2^_2_(8) is built across inversion centres through N—H···O hydrogen bonds. *R*^2^_2_(7) is edge fused with a *R*^2^_1_(6) motif, in which the N—H···N bond is the shared edge, while C—H···O and N—H···O, respectively are their respective characteristic units. An interesting feature is that all these fundamental simple graph-set motifs lead to a complex *R*^6^_6_(36) motif across the inversion centres. Also two significant C—H···π*viz*. C6A—H63A···*Cg*1(C20A→C25A) and C6B—H62B···*Cg*2 (C20B→C25B) interactions are observed. Thus, the presence of a variety of interaction patterns in the crystal structure of a geometrically unfathomable molecule may well be regarded significant in the context of crystal structure prediction.

### Refinement

H atoms were positioned geometrically and refined using a riding model with C—H = 0.95–0.99 Å and with *U*_iso_(H) = 1.2 (1.5 for methyl groups) times *U*_eq_(C).

### Crystal data

**Table:**

C_26_H_29_N_3_O_2_	*Z* = 4
*M**_r_* = 415.52	*F*(000) = 888
Triclinic, *P*1	*D*_x_ = 1.250 Mg m^−^^3^
Hall symbol: -P 1	Mo *K*α radiation, λ = 0.71073 Å
*a* = 8.7516 (4) Å	Cell parameters from 5520 reflections
*b* = 12.4649 (5) Å	θ = 2.4–23.9°
*c* = 21.4605 (8) Å	µ = 0.08 mm^−^^1^
α = 97.654 (2)°	*T* = 293 K
β = 101.024 (2)°	Block, colourless
γ = 102.345 (2)°	0.28 × 0.19 × 0.19 mm
*V* = 2207.45 (16) Å^3^	

### Data collection

**Table:**

Bruker Kappa APEXII CCD diffractometer	7765 independent reflections
Radiation source: fine-focus sealed tube	5493 reflections with *I* > 2σ(*I*)
Graphite monochromator	*R*_int_ = 0.037
ω and φ scan	θ_max_ = 25.0°, θ_min_ = 1.8°
Absorption correction: multi-scan (*SADABS*; Bruker, 2009)	*h* = −10→10
*T*_min_ = 0.93, *T*_max_ = 0.96	*k* = −14→14
44464 measured reflections	*l* = −25→25

### Refinement

**Table:**

Refinement on *F*^2^	Secondary atom site location: difference Fourier map
Least-squares matrix: full	Hydrogen site location: inferred from neighbouring sites
*R*[*F*^2^ > 2σ(*F*^2^)] = 0.040	H-atom parameters constrained
*wR*(*F*^2^) = 0.110	*w* = 1/[σ^2^(*F*_o_^2^) + (0.0495*P*)^2^ + 0.5445*P*] where *P* = (*F*_o_^2^ + 2*F*_c_^2^)/3
*S* = 1.02	(Δ/σ)_max_ < 0.001
7765 reflections	Δρ_max_ = 0.21 e Å^−^^3^
574 parameters	Δρ_min_ = −0.17 e Å^−^^3^
0 restraints	Extinction correction: *SHELXL97* (Sheldrick, 2008), Fc^*^=kFc[1+0.001xFc^2^λ^3^/sin(2θ)]^-1/4^
Primary atom site location: structure-invariant direct methods	Extinction coefficient: 0.0037 (7)

### Special details

**Table:**

Geometry. All e.s.d.'s (except the e.s.d. in the dihedral angle between two l.s. planes) are estimated using the full covariance matrix. The cell e.s.d.'s are taken into account individually in the estimation of e.s.d.'s in distances, angles and torsion angles; correlations between e.s.d.'s in cell parameters are only used when they are defined by crystal symmetry. An approximate (isotropic) treatment of cell e.s.d.'s is used for estimating e.s.d.'s involving l.s. planes.
Refinement. Refinement of *F*^2^ against ALL reflections. The weighted *R*-factor *wR* and goodness of fit *S* are based on *F*^2^, conventional *R*-factors *R* are based on *F*, with *F* set to zero for negative *F*^2^. The threshold expression of *F*^2^ > σ(*F*^2^) is used only for calculating *R*-factors(gt) *etc*. and is not relevant to the choice of reflections for refinement. *R*-factors based on *F*^2^ are statistically about twice as large as those based on *F*, and *R*- factors based on ALL data will be even larger.

### Fractional atomic coordinates and isotropic or equivalent isotropic displacement parameters (Å^2^)

**Table:**

	*x*	*y*	*z*	*U*_iso_*/*U*_eq_	Occ. (<1)
O1A	0.94742 (14)	0.97304 (9)	0.41175 (5)	0.0410 (3)	
O2A	0.66024 (15)	0.52962 (10)	0.36307 (6)	0.0506 (3)	
N1A	0.98111 (15)	0.80851 (10)	0.31517 (6)	0.0330 (3)	
N2A	1.02786 (17)	0.86038 (11)	0.48069 (6)	0.0432 (4)	
H2A	1.0432	0.9046	0.5169	0.052*	
N3A	0.63813 (17)	0.84523 (12)	0.41103 (7)	0.0446 (4)	
C1A	0.92833 (18)	0.77439 (12)	0.37159 (7)	0.0309 (4)	
C2A	0.74340 (18)	0.71642 (12)	0.34351 (7)	0.0308 (4)	
C3A	0.74369 (18)	0.66023 (12)	0.27416 (7)	0.0315 (4)	
H3A	0.7765	0.5906	0.2781	0.038*	
C4A	0.87877 (19)	0.73809 (13)	0.25463 (7)	0.0334 (4)	
H4A	0.8326	0.7858	0.2275	0.040*	
C5A	0.9919 (2)	0.68726 (16)	0.22146 (9)	0.0507 (5)	
H51A	0.9543	0.6724	0.1749	0.061*	
H52A	1.0057	0.6187	0.2359	0.061*	
C6AA	1.1467 (3)	0.7797 (3)	0.24341 (15)	0.0532 (11)	0.706 (7)
H61A	1.2395	0.7507	0.2394	0.064*	0.706 (7)
H62A	1.1437	0.8390	0.2186	0.064*	0.706 (7)
C6A	1.1412 (9)	0.7035 (9)	0.2653 (4)	0.067 (3)	0.294 (7)
H63A	1.2291	0.7116	0.2434	0.080*	0.294 (7)
H64A	1.1424	0.6425	0.2889	0.080*	0.294 (7)
C7A	1.1497 (2)	0.81995 (17)	0.31269 (9)	0.0504 (5)	
H71A	1.1983	0.7749	0.3403	0.061*	
H72A	1.2100	0.8972	0.3265	0.061*	
C8A	0.96279 (19)	0.88168 (13)	0.42297 (8)	0.0345 (4)	
C9A	1.0674 (2)	0.75700 (14)	0.47431 (8)	0.0397 (4)	
C10A	1.1479 (2)	0.71193 (16)	0.52181 (9)	0.0541 (5)	
H10A	1.1798	0.7491	0.5645	0.065*	
C11A	1.1795 (3)	0.60970 (18)	0.50390 (11)	0.0608 (6)	
H11A	1.2346	0.5777	0.5349	0.073*	
C12A	1.1307 (2)	0.55462 (16)	0.44085 (10)	0.0555 (5)	
H12A	1.1536	0.4860	0.4298	0.067*	
C13A	1.0476 (2)	0.60007 (14)	0.39347 (9)	0.0441 (4)	
H13A	1.0135	0.5620	0.3510	0.053*	
C14A	1.01634 (19)	0.70280 (13)	0.41043 (8)	0.0349 (4)	
C15A	0.67971 (19)	0.62814 (14)	0.38218 (8)	0.0374 (4)	
C16A	0.6375 (3)	0.66840 (17)	0.44407 (9)	0.0542 (5)	
H16A	0.7352	0.6965	0.4776	0.065*	
H16B	0.5711	0.6061	0.4569	0.065*	
C17A	0.5490 (2)	0.75934 (18)	0.43833 (10)	0.0583 (5)	
H17A	0.4425	0.7287	0.4109	0.070*	
H17B	0.5379	0.7908	0.4806	0.070*	
C18A	0.6423 (2)	0.80318 (14)	0.34508 (8)	0.0378 (4)	
H18A	0.6879	0.8647	0.3256	0.045*	
H18B	0.5338	0.7692	0.3202	0.045*	
C19A	0.5855 (3)	0.94882 (17)	0.41722 (10)	0.0622 (6)	
H19A	0.4759	0.9348	0.3940	0.093*	
H19B	0.6522	1.0032	0.3998	0.093*	
H19C	0.5938	0.9766	0.4620	0.093*	
C20A	0.58431 (19)	0.62975 (13)	0.22520 (7)	0.0334 (4)	
C21A	0.4860 (2)	0.52211 (15)	0.21288 (9)	0.0485 (5)	
H21A	0.5171	0.4692	0.2359	0.058*	
C22A	0.3429 (2)	0.49259 (17)	0.16709 (9)	0.0571 (5)	
H22A	0.2795	0.4201	0.1601	0.068*	
C23A	0.2911 (2)	0.56729 (17)	0.13138 (9)	0.0504 (5)	
C24A	0.3886 (2)	0.67381 (16)	0.14344 (9)	0.0476 (5)	
H24A	0.3572	0.7263	0.1201	0.057*	
C25A	0.5316 (2)	0.70464 (14)	0.18928 (8)	0.0417 (4)	
H25A	0.5941	0.7774	0.1962	0.050*	
C26A	0.1373 (3)	0.5351 (2)	0.08001 (11)	0.0786 (7)	
H26A	0.1455	0.4799	0.0459	0.118*	
H26B	0.1191	0.5999	0.0630	0.118*	
H26C	0.0494	0.5049	0.0984	0.118*	
O1B	0.80365 (16)	−0.04225 (10)	0.18075 (6)	0.0539 (3)	
O2B	0.8647 (2)	0.41105 (11)	0.22578 (7)	0.0696 (4)	
N1B	1.01620 (19)	0.16146 (13)	0.15026 (7)	0.0503 (4)	
N2B	0.97215 (18)	0.03574 (12)	0.27920 (7)	0.0473 (4)	
H2B	0.9583	−0.0217	0.2976	0.057*	
N3B	0.61912 (19)	0.10803 (13)	0.24203 (8)	0.0528 (4)	
C1B	0.9443 (2)	0.15839 (13)	0.20515 (8)	0.0398 (4)	
C2B	0.7965 (2)	0.21432 (13)	0.18396 (8)	0.0393 (4)	
C3B	0.8158 (2)	0.24387 (14)	0.11669 (8)	0.0439 (4)	
H3B	0.8938	0.3164	0.1254	0.053*	
C4B	0.8991 (2)	0.15759 (15)	0.09240 (8)	0.0472 (5)	
H4B	0.8242	0.0837	0.0790	0.057*	
C5B	1.0059 (3)	0.1781 (2)	0.04515 (11)	0.0700 (6)	
H51B	1.0456	0.2574	0.0465	0.084*	
H52B	0.9485	0.1429	0.0014	0.084*	
C6B	1.1438 (3)	0.1248 (2)	0.06857 (12)	0.0817 (7)	
H61B	1.1364	0.0572	0.0387	0.098*	
H62B	1.2467	0.1762	0.0721	0.098*	
C7B	1.1265 (3)	0.0981 (2)	0.13420 (11)	0.0717 (6)	
H71B	1.0829	0.0188	0.1317	0.086*	
H72B	1.2286	0.1220	0.1654	0.086*	
C8B	0.8908 (2)	0.03814 (14)	0.21913 (9)	0.0417 (4)	
C9B	1.0814 (2)	0.13785 (15)	0.30764 (9)	0.0437 (4)	
C10B	1.1940 (2)	0.16449 (18)	0.36528 (9)	0.0579 (5)	
H10B	1.2000	0.1138	0.3932	0.069*	
C11B	1.2979 (3)	0.26945 (19)	0.37998 (10)	0.0642 (6)	
H11B	1.3756	0.2893	0.4185	0.077*	
C12B	1.2894 (3)	0.34531 (18)	0.33923 (10)	0.0626 (6)	
H12B	1.3612	0.4152	0.3503	0.075*	
C13B	1.1745 (2)	0.31804 (15)	0.28186 (10)	0.0546 (5)	
H13B	1.1684	0.3692	0.2542	0.065*	
C14B	1.0691 (2)	0.21393 (14)	0.26617 (8)	0.0421 (4)	
C15B	0.8128 (2)	0.31757 (15)	0.23421 (9)	0.0486 (5)	
C16B	0.7613 (3)	0.29696 (18)	0.29528 (10)	0.0628 (6)	
H16C	0.7453	0.3656	0.3171	0.075*	
H16D	0.8466	0.2765	0.3236	0.075*	
C17B	0.6081 (3)	0.20570 (19)	0.28375 (10)	0.0658 (6)	
H17C	0.5909	0.1865	0.3246	0.079*	
H17D	0.5174	0.2323	0.2640	0.079*	
C18B	0.6336 (2)	0.13550 (15)	0.17934 (9)	0.0457 (4)	
H18C	0.5488	0.1706	0.1634	0.055*	
H18D	0.6213	0.0675	0.1490	0.055*	
C19B	0.4850 (3)	0.0124 (2)	0.23642 (13)	0.0766 (7)	
H19D	0.4818	−0.0053	0.2784	0.115*	
H19E	0.4984	−0.0506	0.2089	0.115*	
H19F	0.3864	0.0302	0.2182	0.115*	
C20B	0.6676 (2)	0.25512 (15)	0.07119 (9)	0.0481 (5)	
C21B	0.6182 (3)	0.35393 (18)	0.07883 (10)	0.0635 (6)	
H21B	0.6741	0.4114	0.1131	0.076*	
C22B	0.4870 (3)	0.3677 (2)	0.03610 (11)	0.0699 (6)	
H22B	0.4559	0.4344	0.0424	0.084*	
C23B	0.4009 (3)	0.2852 (2)	−0.01565 (10)	0.0628 (6)	
C24B	0.4502 (3)	0.1881 (2)	−0.02321 (10)	0.0642 (6)	
H24B	0.3947	0.1311	−0.0578	0.077*	
C25B	0.5807 (3)	0.17275 (17)	0.01928 (9)	0.0569 (5)	
H25B	0.6107	0.1056	0.0128	0.068*	
C26B	0.2593 (3)	0.3020 (3)	−0.06182 (11)	0.0868 (8)	
H26D	0.1886	0.3289	−0.0380	0.130*	
H26E	0.2029	0.2323	−0.0897	0.130*	
H26F	0.2961	0.3556	−0.0873	0.130*	

### Atomic displacement parameters (Å^2^)

**Table:**

	*U*^11^	*U*^22^	*U*^33^	*U*^12^	*U*^13^	*U*^23^
O1A	0.0532 (8)	0.0308 (6)	0.0359 (7)	0.0096 (5)	0.0057 (6)	0.0029 (5)
O2A	0.0578 (8)	0.0402 (7)	0.0510 (8)	0.0025 (6)	0.0136 (6)	0.0127 (6)
N1A	0.0292 (7)	0.0360 (7)	0.0295 (7)	0.0022 (6)	0.0042 (6)	0.0038 (6)
N2A	0.0561 (10)	0.0402 (8)	0.0268 (8)	0.0130 (7)	−0.0021 (7)	−0.0016 (6)
N3A	0.0440 (9)	0.0538 (9)	0.0367 (8)	0.0180 (7)	0.0110 (7)	−0.0018 (7)
C1A	0.0315 (9)	0.0302 (8)	0.0280 (8)	0.0063 (7)	0.0028 (7)	0.0027 (6)
C2A	0.0308 (9)	0.0327 (8)	0.0263 (8)	0.0063 (7)	0.0042 (7)	0.0026 (6)
C3A	0.0339 (9)	0.0295 (8)	0.0290 (8)	0.0063 (7)	0.0049 (7)	0.0036 (6)
C4A	0.0334 (9)	0.0357 (9)	0.0284 (9)	0.0061 (7)	0.0036 (7)	0.0051 (7)
C5A	0.0517 (12)	0.0541 (11)	0.0456 (11)	0.0101 (9)	0.0190 (10)	−0.0009 (9)
C6AA	0.0420 (17)	0.064 (2)	0.057 (2)	0.0122 (14)	0.0213 (14)	0.0091 (16)
C6A	0.057 (5)	0.109 (8)	0.055 (5)	0.044 (5)	0.027 (4)	0.026 (5)
C7A	0.0315 (10)	0.0657 (12)	0.0488 (12)	0.0039 (9)	0.0061 (9)	0.0093 (9)
C8A	0.0348 (9)	0.0330 (9)	0.0321 (9)	0.0057 (7)	0.0035 (7)	0.0039 (7)
C9A	0.0404 (10)	0.0392 (9)	0.0367 (10)	0.0087 (8)	0.0022 (8)	0.0090 (8)
C10A	0.0615 (13)	0.0588 (12)	0.0382 (11)	0.0153 (10)	−0.0020 (9)	0.0143 (9)
C11A	0.0616 (14)	0.0635 (13)	0.0639 (14)	0.0240 (11)	0.0055 (11)	0.0327 (11)
C12A	0.0588 (13)	0.0443 (11)	0.0685 (14)	0.0218 (9)	0.0111 (11)	0.0186 (10)
C13A	0.0450 (11)	0.0382 (9)	0.0490 (11)	0.0141 (8)	0.0063 (9)	0.0072 (8)
C14A	0.0321 (9)	0.0342 (9)	0.0366 (10)	0.0064 (7)	0.0042 (7)	0.0087 (7)
C15A	0.0313 (9)	0.0432 (10)	0.0339 (9)	0.0041 (8)	0.0030 (7)	0.0084 (8)
C16A	0.0607 (13)	0.0633 (12)	0.0406 (11)	0.0083 (10)	0.0200 (10)	0.0153 (9)
C17A	0.0533 (13)	0.0798 (14)	0.0455 (12)	0.0173 (11)	0.0223 (10)	0.0062 (10)
C18A	0.0345 (10)	0.0432 (9)	0.0338 (9)	0.0121 (8)	0.0034 (7)	0.0023 (7)
C19A	0.0554 (13)	0.0682 (13)	0.0611 (13)	0.0292 (11)	0.0084 (10)	−0.0110 (10)
C20A	0.0337 (9)	0.0374 (9)	0.0253 (8)	0.0050 (7)	0.0063 (7)	−0.0006 (7)
C21A	0.0512 (12)	0.0430 (10)	0.0397 (10)	−0.0021 (9)	−0.0008 (9)	0.0056 (8)
C22A	0.0513 (12)	0.0530 (12)	0.0473 (12)	−0.0114 (9)	−0.0002 (10)	−0.0034 (9)
C23A	0.0408 (11)	0.0656 (13)	0.0359 (10)	0.0077 (10)	0.0033 (8)	−0.0049 (9)
C24A	0.0416 (11)	0.0628 (12)	0.0381 (10)	0.0166 (9)	0.0042 (9)	0.0091 (9)
C25A	0.0381 (10)	0.0425 (10)	0.0412 (10)	0.0073 (8)	0.0052 (8)	0.0060 (8)
C26A	0.0538 (14)	0.0992 (18)	0.0604 (15)	0.0087 (13)	−0.0141 (11)	−0.0078 (13)
O1B	0.0582 (9)	0.0345 (7)	0.0584 (8)	0.0059 (6)	−0.0040 (7)	0.0056 (6)
O2B	0.1059 (13)	0.0372 (8)	0.0651 (10)	0.0171 (8)	0.0196 (9)	0.0074 (7)
N1B	0.0529 (10)	0.0583 (10)	0.0469 (9)	0.0208 (8)	0.0168 (8)	0.0150 (7)
N2B	0.0500 (9)	0.0386 (8)	0.0484 (9)	0.0038 (7)	0.0005 (7)	0.0179 (7)
N3B	0.0490 (10)	0.0617 (10)	0.0578 (10)	0.0179 (8)	0.0191 (8)	0.0291 (8)
C1B	0.0439 (10)	0.0350 (9)	0.0410 (10)	0.0079 (8)	0.0101 (8)	0.0112 (8)
C2B	0.0479 (11)	0.0342 (9)	0.0373 (10)	0.0120 (8)	0.0086 (8)	0.0102 (7)
C3B	0.0583 (12)	0.0354 (9)	0.0398 (10)	0.0108 (8)	0.0121 (9)	0.0123 (8)
C4B	0.0592 (12)	0.0426 (10)	0.0416 (11)	0.0130 (9)	0.0130 (9)	0.0104 (8)
C5B	0.0937 (18)	0.0727 (14)	0.0555 (13)	0.0257 (13)	0.0365 (13)	0.0159 (11)
C6B	0.0813 (18)	0.1004 (19)	0.0769 (17)	0.0328 (15)	0.0398 (14)	0.0149 (14)
C7B	0.0665 (15)	0.0865 (16)	0.0767 (16)	0.0340 (13)	0.0300 (13)	0.0213 (13)
C8B	0.0415 (11)	0.0352 (9)	0.0469 (11)	0.0091 (8)	0.0053 (9)	0.0095 (8)
C9B	0.0399 (10)	0.0464 (10)	0.0422 (11)	0.0049 (8)	0.0079 (8)	0.0103 (8)
C10B	0.0530 (13)	0.0693 (14)	0.0446 (12)	0.0042 (11)	0.0040 (10)	0.0145 (10)
C11B	0.0521 (13)	0.0817 (15)	0.0444 (12)	−0.0024 (11)	0.0055 (10)	−0.0002 (11)
C12B	0.0544 (13)	0.0588 (13)	0.0595 (14)	−0.0108 (10)	0.0143 (11)	−0.0039 (11)
C13B	0.0550 (13)	0.0455 (11)	0.0582 (13)	−0.0009 (9)	0.0154 (11)	0.0099 (9)
C14B	0.0400 (10)	0.0397 (10)	0.0450 (11)	0.0048 (8)	0.0108 (8)	0.0083 (8)
C15B	0.0585 (12)	0.0419 (11)	0.0464 (11)	0.0181 (9)	0.0076 (9)	0.0081 (8)
C16B	0.0859 (17)	0.0659 (13)	0.0453 (12)	0.0325 (13)	0.0209 (11)	0.0093 (10)
C17B	0.0719 (16)	0.0869 (16)	0.0587 (14)	0.0377 (13)	0.0309 (12)	0.0301 (12)
C18B	0.0459 (11)	0.0479 (10)	0.0472 (11)	0.0151 (9)	0.0088 (9)	0.0184 (9)
C19B	0.0526 (14)	0.0855 (16)	0.1029 (19)	0.0141 (12)	0.0219 (13)	0.0549 (15)
C20B	0.0634 (13)	0.0493 (11)	0.0388 (10)	0.0196 (9)	0.0148 (9)	0.0188 (9)
C21B	0.0896 (17)	0.0579 (12)	0.0470 (12)	0.0306 (12)	0.0081 (11)	0.0134 (10)
C22B	0.0951 (18)	0.0806 (16)	0.0556 (14)	0.0522 (14)	0.0231 (13)	0.0296 (13)
C23B	0.0687 (15)	0.0930 (17)	0.0403 (12)	0.0336 (13)	0.0196 (11)	0.0271 (12)
C24B	0.0677 (15)	0.0778 (15)	0.0450 (12)	0.0162 (12)	0.0088 (11)	0.0121 (11)
C25B	0.0693 (14)	0.0551 (12)	0.0467 (12)	0.0189 (11)	0.0087 (10)	0.0119 (10)
C26B	0.0811 (18)	0.140 (2)	0.0568 (15)	0.0522 (17)	0.0172 (13)	0.0352 (15)

### Geometric parameters (Å, º)

**Table:**

O1A—C8A	1.2244 (18)	C26A—H26B	0.9600
O2A—C15A	1.2062 (19)	C26A—H26C	0.9600
N1A—C1A	1.459 (2)	O1B—C8B	1.215 (2)
N1A—C7A	1.464 (2)	O2B—C15B	1.208 (2)
N1A—C4A	1.475 (2)	N1B—C7B	1.433 (3)
N2A—C8A	1.346 (2)	N1B—C4B	1.439 (2)
N2A—C9A	1.400 (2)	N1B—C1B	1.439 (2)
N2A—H2A	0.8600	N2B—C8B	1.356 (2)
N3A—C17A	1.443 (2)	N2B—C9B	1.398 (2)
N3A—C18A	1.452 (2)	N2B—H2B	0.8600
N3A—C19A	1.459 (2)	N3B—C17B	1.444 (3)
C1A—C14A	1.527 (2)	N3B—C18B	1.454 (2)
C1A—C8A	1.550 (2)	N3B—C19B	1.457 (3)
C1A—C2A	1.586 (2)	C1B—C14B	1.508 (2)
C2A—C18A	1.538 (2)	C1B—C8B	1.559 (2)
C2A—C15A	1.539 (2)	C1B—C2B	1.616 (2)
C2A—C3A	1.559 (2)	C2B—C18B	1.526 (2)
C3A—C20A	1.517 (2)	C2B—C15B	1.527 (2)
C3A—C4A	1.524 (2)	C2B—C3B	1.567 (2)
C3A—H3A	0.9800	C3B—C20B	1.512 (3)
C4A—C5A	1.519 (2)	C3B—C4B	1.514 (2)
C4A—H4A	0.9800	C3B—H3B	0.9800
C5A—C6A	1.414 (8)	C4B—C5B	1.516 (3)
C5A—C6AA	1.526 (3)	C4B—H4B	0.9800
C5A—H51A	0.9700	C5B—C6B	1.533 (3)
C5A—H52A	0.9700	C5B—H51B	0.9700
C6AA—C7A	1.498 (3)	C5B—H52B	0.9700
C6AA—H61A	0.9700	C6B—C7B	1.517 (3)
C6AA—H62A	0.9700	C6B—H61B	0.9700
C6A—C7A	1.637 (9)	C6B—H62B	0.9700
C6A—H63A	0.9700	C7B—H71B	0.9700
C6A—H64A	0.9700	C7B—H72B	0.9700
C7A—H71A	0.9700	C9B—C10B	1.377 (3)
C7A—H72A	0.9700	C9B—C14B	1.391 (2)
C9A—C10A	1.378 (2)	C10B—C11B	1.382 (3)
C9A—C14A	1.387 (2)	C10B—H10B	0.9300
C10A—C11A	1.382 (3)	C11B—C12B	1.375 (3)
C10A—H10A	0.9300	C11B—H11B	0.9300
C11A—C12A	1.375 (3)	C12B—C13B	1.383 (3)
C11A—H11A	0.9300	C12B—H12B	0.9300
C12A—C13A	1.389 (2)	C13B—C14B	1.379 (2)
C12A—H12A	0.9300	C13B—H13B	0.9300
C13A—C14A	1.382 (2)	C15B—C16B	1.501 (3)
C13A—H13A	0.9300	C16B—C17B	1.516 (3)
C15A—C16A	1.497 (2)	C16B—H16C	0.9700
C16A—C17A	1.509 (3)	C16B—H16D	0.9700
C16A—H16A	0.9700	C17B—H17C	0.9700
C16A—H16B	0.9700	C17B—H17D	0.9700
C17A—H17A	0.9700	C18B—H18C	0.9700
C17A—H17B	0.9700	C18B—H18D	0.9700
C18A—H18A	0.9700	C19B—H19D	0.9600
C18A—H18B	0.9700	C19B—H19E	0.9600
C19A—H19A	0.9600	C19B—H19F	0.9600
C19A—H19B	0.9600	C20B—C25B	1.382 (3)
C19A—H19C	0.9600	C20B—C21B	1.390 (3)
C20A—C25A	1.385 (2)	C21B—C22B	1.383 (3)
C20A—C21A	1.391 (2)	C21B—H21B	0.9300
C21A—C22A	1.380 (3)	C22B—C23B	1.379 (3)
C21A—H21A	0.9300	C22B—H22B	0.9300
C22A—C23A	1.377 (3)	C23B—C24B	1.369 (3)
C22A—H22A	0.9300	C23B—C26B	1.503 (3)
C23A—C24A	1.378 (3)	C24B—C25B	1.384 (3)
C23A—C26A	1.507 (3)	C24B—H24B	0.9300
C24A—C25A	1.380 (2)	C25B—H25B	0.9300
C24A—H24A	0.9300	C26B—H26D	0.9600
C25A—H25A	0.9300	C26B—H26E	0.9600
C26A—H26A	0.9600	C26B—H26F	0.9600
			
C1A—N1A—C7A	118.87 (13)	C23A—C26A—H26A	109.5
C1A—N1A—C4A	111.65 (12)	C23A—C26A—H26B	109.5
C7A—N1A—C4A	108.84 (13)	H26A—C26A—H26B	109.5
C8A—N2A—C9A	111.37 (14)	C23A—C26A—H26C	109.5
C8A—N2A—H2A	124.3	H26A—C26A—H26C	109.5
C9A—N2A—H2A	124.3	H26B—C26A—H26C	109.5
C17A—N3A—C18A	111.02 (14)	C7B—N1B—C4B	108.59 (16)
C17A—N3A—C19A	113.69 (16)	C7B—N1B—C1B	126.19 (15)
C18A—N3A—C19A	112.65 (14)	C4B—N1B—C1B	111.17 (14)
N1A—C1A—C14A	119.65 (13)	C8B—N2B—C9B	111.89 (14)
N1A—C1A—C8A	106.99 (12)	C8B—N2B—H2B	124.1
C14A—C1A—C8A	100.53 (12)	C9B—N2B—H2B	124.1
N1A—C1A—C2A	102.75 (11)	C17B—N3B—C18B	109.34 (15)
C14A—C1A—C2A	112.81 (12)	C17B—N3B—C19B	112.47 (17)
C8A—C1A—C2A	114.51 (13)	C18B—N3B—C19B	111.71 (17)
C18A—C2A—C15A	107.69 (13)	N1B—C1B—C14B	109.95 (14)
C18A—C2A—C3A	114.06 (12)	N1B—C1B—C8B	113.05 (14)
C15A—C2A—C3A	110.71 (12)	C14B—C1B—C8B	100.99 (13)
C18A—C2A—C1A	110.80 (12)	N1B—C1B—C2B	101.40 (13)
C15A—C2A—C1A	112.00 (12)	C14B—C1B—C2B	118.35 (14)
C3A—C2A—C1A	101.60 (12)	C8B—C1B—C2B	113.55 (14)
C20A—C3A—C4A	114.31 (13)	C18B—C2B—C15B	107.40 (15)
C20A—C3A—C2A	116.31 (13)	C18B—C2B—C3B	111.75 (14)
C4A—C3A—C2A	104.97 (12)	C15B—C2B—C3B	112.54 (13)
C20A—C3A—H3A	106.9	C18B—C2B—C1B	112.55 (13)
C4A—C3A—H3A	106.9	C15B—C2B—C1B	109.27 (14)
C2A—C3A—H3A	106.9	C3B—C2B—C1B	103.38 (13)
N1A—C4A—C5A	105.27 (13)	C20B—C3B—C4B	116.77 (15)
N1A—C4A—C3A	105.94 (12)	C20B—C3B—C2B	117.33 (15)
C5A—C4A—C3A	118.54 (14)	C4B—C3B—C2B	102.11 (13)
N1A—C4A—H4A	108.9	C20B—C3B—H3B	106.6
C5A—C4A—H4A	108.9	C4B—C3B—H3B	106.6
C3A—C4A—H4A	108.9	C2B—C3B—H3B	106.6
C6A—C5A—C4A	109.8 (3)	N1B—C4B—C3B	100.22 (14)
C4A—C5A—C6AA	101.44 (16)	N1B—C4B—C5B	100.92 (16)
C6A—C5A—H51A	135.8	C3B—C4B—C5B	122.04 (15)
C4A—C5A—H51A	111.5	N1B—C4B—H4B	110.8
C6AA—C5A—H51A	111.5	C3B—C4B—H4B	110.8
C6A—C5A—H52A	68.2	C5B—C4B—H4B	110.8
C4A—C5A—H52A	111.5	C4B—C5B—C6B	103.76 (17)
C6AA—C5A—H52A	111.5	C4B—C5B—H51B	111.0
H51A—C5A—H52A	109.3	C6B—C5B—H51B	111.0
C7A—C6AA—C5A	103.1 (2)	C4B—C5B—H52B	111.0
C7A—C6AA—H61A	111.2	C6B—C5B—H52B	111.0
C5A—C6AA—H61A	111.2	H51B—C5B—H52B	109.0
C7A—C6AA—H62A	111.2	C7B—C6B—C5B	106.14 (18)
C5A—C6AA—H62A	111.2	C7B—C6B—H61B	110.5
H61A—C6AA—H62A	109.1	C5B—C6B—H61B	110.5
C5A—C6A—C7A	101.6 (5)	C7B—C6B—H62B	110.5
C5A—C6A—H63A	111.5	C5B—C6B—H62B	110.5
C7A—C6A—H63A	111.5	H61B—C6B—H62B	108.7
C5A—C6A—H64A	111.5	N1B—C7B—C6B	102.28 (18)
C7A—C6A—H64A	111.5	N1B—C7B—H71B	111.3
H63A—C6A—H64A	109.3	C6B—C7B—H71B	111.3
N1A—C7A—C6AA	105.09 (16)	N1B—C7B—H72B	111.3
N1A—C7A—C6A	101.9 (3)	C6B—C7B—H72B	111.3
N1A—C7A—H71A	110.7	H71B—C7B—H72B	109.2
C6AA—C7A—H71A	110.7	O1B—C8B—N2B	125.57 (15)
C6A—C7A—H71A	74.1	O1B—C8B—C1B	125.97 (15)
N1A—C7A—H72A	110.7	N2B—C8B—C1B	108.10 (14)
C6AA—C7A—H72A	110.7	C10B—C9B—C14B	121.75 (17)
C6A—C7A—H72A	142.8	C10B—C9B—N2B	128.76 (16)
H71A—C7A—H72A	108.8	C14B—C9B—N2B	109.38 (15)
O1A—C8A—N2A	125.70 (15)	C9B—C10B—C11B	117.42 (18)
O1A—C8A—C1A	125.42 (14)	C9B—C10B—H10B	121.3
N2A—C8A—C1A	108.55 (13)	C11B—C10B—H10B	121.3
C10A—C9A—C14A	122.37 (16)	C12B—C11B—C10B	121.77 (19)
C10A—C9A—N2A	127.68 (16)	C12B—C11B—H11B	119.1
C14A—C9A—N2A	109.93 (13)	C10B—C11B—H11B	119.1
C9A—C10A—C11A	117.75 (18)	C11B—C12B—C13B	120.26 (19)
C9A—C10A—H10A	121.1	C11B—C12B—H12B	119.9
C11A—C10A—H10A	121.1	C13B—C12B—H12B	119.9
C12A—C11A—C10A	120.92 (17)	C14B—C13B—C12B	119.10 (18)
C12A—C11A—H11A	119.5	C14B—C13B—H13B	120.5
C10A—C11A—H11A	119.5	C12B—C13B—H13B	120.5
C11A—C12A—C13A	120.87 (17)	C13B—C14B—C9B	119.69 (17)
C11A—C12A—H12A	119.6	C13B—C14B—C1B	130.42 (16)
C13A—C12A—H12A	119.6	C9B—C14B—C1B	109.56 (14)
C14A—C13A—C12A	118.96 (17)	O2B—C15B—C16B	121.35 (17)
C14A—C13A—H13A	120.5	O2B—C15B—C2B	122.23 (17)
C12A—C13A—H13A	120.5	C16B—C15B—C2B	116.42 (16)
C13A—C14A—C9A	119.12 (15)	C15B—C16B—C17B	113.12 (17)
C13A—C14A—C1A	132.36 (15)	C15B—C16B—H16C	109.0
C9A—C14A—C1A	108.51 (13)	C17B—C16B—H16C	109.0
O2A—C15A—C16A	120.55 (15)	C15B—C16B—H16D	109.0
O2A—C15A—C2A	121.80 (15)	C17B—C16B—H16D	109.0
C16A—C15A—C2A	117.62 (15)	H16C—C16B—H16D	107.8
C15A—C16A—C17A	112.37 (15)	N3B—C17B—C16B	110.04 (17)
C15A—C16A—H16A	109.1	N3B—C17B—H17C	109.7
C17A—C16A—H16A	109.1	C16B—C17B—H17C	109.7
C15A—C16A—H16B	109.1	N3B—C17B—H17D	109.7
C17A—C16A—H16B	109.1	C16B—C17B—H17D	109.7
H16A—C16A—H16B	107.9	H17C—C17B—H17D	108.2
N3A—C17A—C16A	108.98 (15)	N3B—C18B—C2B	110.83 (14)
N3A—C17A—H17A	109.9	N3B—C18B—H18C	109.5
C16A—C17A—H17A	109.9	C2B—C18B—H18C	109.5
N3A—C17A—H17B	109.9	N3B—C18B—H18D	109.5
C16A—C17A—H17B	109.9	C2B—C18B—H18D	109.5
H17A—C17A—H17B	108.3	H18C—C18B—H18D	108.1
N3A—C18A—C2A	110.66 (13)	N3B—C19B—H19D	109.5
N3A—C18A—H18A	109.5	N3B—C19B—H19E	109.5
C2A—C18A—H18A	109.5	H19D—C19B—H19E	109.5
N3A—C18A—H18B	109.5	N3B—C19B—H19F	109.5
C2A—C18A—H18B	109.5	H19D—C19B—H19F	109.5
H18A—C18A—H18B	108.1	H19E—C19B—H19F	109.5
N3A—C19A—H19A	109.5	C25B—C20B—C21B	117.16 (18)
N3A—C19A—H19B	109.5	C25B—C20B—C3B	122.95 (17)
H19A—C19A—H19B	109.5	C21B—C20B—C3B	119.81 (18)
N3A—C19A—H19C	109.5	C22B—C21B—C20B	120.7 (2)
H19A—C19A—H19C	109.5	C22B—C21B—H21B	119.6
H19B—C19A—H19C	109.5	C20B—C21B—H21B	119.6
C25A—C20A—C21A	116.76 (15)	C23B—C22B—C21B	121.8 (2)
C25A—C20A—C3A	122.63 (14)	C23B—C22B—H22B	119.1
C21A—C20A—C3A	120.58 (14)	C21B—C22B—H22B	119.1
C22A—C21A—C20A	121.06 (17)	C24B—C23B—C22B	117.3 (2)
C22A—C21A—H21A	119.5	C24B—C23B—C26B	121.6 (2)
C20A—C21A—H21A	119.5	C22B—C23B—C26B	121.0 (2)
C23A—C22A—C21A	121.99 (18)	C23B—C24B—C25B	121.6 (2)
C23A—C22A—H22A	119.0	C23B—C24B—H24B	119.2
C21A—C22A—H22A	119.0	C25B—C24B—H24B	119.2
C22A—C23A—C24A	116.99 (17)	C20B—C25B—C24B	121.4 (2)
C22A—C23A—C26A	122.42 (19)	C20B—C25B—H25B	119.3
C24A—C23A—C26A	120.57 (19)	C24B—C25B—H25B	119.3
C23A—C24A—C25A	121.64 (17)	C23B—C26B—H26D	109.5
C23A—C24A—H24A	119.2	C23B—C26B—H26E	109.5
C25A—C24A—H24A	119.2	H26D—C26B—H26E	109.5
C24A—C25A—C20A	121.56 (16)	C23B—C26B—H26F	109.5
C24A—C25A—H25A	119.2	H26D—C26B—H26F	109.5
C20A—C25A—H25A	119.2	H26E—C26B—H26F	109.5
			
C7A—N1A—C1A—C14A	−27.6 (2)	C22A—C23A—C24A—C25A	−0.1 (3)
C4A—N1A—C1A—C14A	100.38 (15)	C26A—C23A—C24A—C25A	−178.62 (19)
C7A—N1A—C1A—C8A	85.58 (16)	C23A—C24A—C25A—C20A	0.3 (3)
C4A—N1A—C1A—C8A	−146.43 (12)	C21A—C20A—C25A—C24A	−0.1 (3)
C7A—N1A—C1A—C2A	−153.49 (13)	C3A—C20A—C25A—C24A	177.76 (16)
C4A—N1A—C1A—C2A	−25.50 (15)	C7B—N1B—C1B—C14B	71.1 (2)
N1A—C1A—C2A—C18A	−86.50 (14)	C4B—N1B—C1B—C14B	−153.96 (14)
C14A—C1A—C2A—C18A	143.31 (13)	C7B—N1B—C1B—C8B	−41.0 (3)
C8A—C1A—C2A—C18A	29.13 (17)	C4B—N1B—C1B—C8B	94.02 (17)
N1A—C1A—C2A—C15A	153.23 (13)	C7B—N1B—C1B—C2B	−162.89 (19)
C14A—C1A—C2A—C15A	23.04 (18)	C4B—N1B—C1B—C2B	−27.91 (17)
C8A—C1A—C2A—C15A	−91.14 (15)	N1B—C1B—C2B—C18B	119.23 (15)
N1A—C1A—C2A—C3A	35.05 (13)	C14B—C1B—C2B—C18B	−120.48 (16)
C14A—C1A—C2A—C3A	−95.14 (14)	C8B—C1B—C2B—C18B	−2.3 (2)
C8A—C1A—C2A—C3A	150.67 (12)	N1B—C1B—C2B—C15B	−121.54 (15)
C18A—C2A—C3A—C20A	−40.86 (18)	C14B—C1B—C2B—C15B	−1.3 (2)
C15A—C2A—C3A—C20A	80.78 (16)	C8B—C1B—C2B—C15B	116.88 (16)
C1A—C2A—C3A—C20A	−160.11 (12)	N1B—C1B—C2B—C3B	−1.51 (16)
C18A—C2A—C3A—C4A	86.55 (15)	C14B—C1B—C2B—C3B	118.78 (15)
C15A—C2A—C3A—C4A	−151.80 (13)	C8B—C1B—C2B—C3B	−123.09 (15)
C1A—C2A—C3A—C4A	−32.70 (14)	C18B—C2B—C3B—C20B	35.3 (2)
C1A—N1A—C4A—C5A	−121.48 (14)	C15B—C2B—C3B—C20B	−85.67 (19)
C7A—N1A—C4A—C5A	11.69 (17)	C1B—C2B—C3B—C20B	156.56 (15)
C1A—N1A—C4A—C3A	4.89 (16)	C18B—C2B—C3B—C4B	−93.74 (17)
C7A—N1A—C4A—C3A	138.07 (13)	C15B—C2B—C3B—C4B	145.32 (15)
C20A—C3A—C4A—N1A	147.06 (13)	C1B—C2B—C3B—C4B	27.55 (16)
C2A—C3A—C4A—N1A	18.44 (15)	C7B—N1B—C4B—C3B	−170.33 (16)
C20A—C3A—C4A—C5A	−95.09 (17)	C1B—N1B—C4B—C3B	46.70 (17)
C2A—C3A—C4A—C5A	136.29 (15)	C7B—N1B—C4B—C5B	−44.6 (2)
N1A—C4A—C5A—C6A	13.3 (5)	C1B—N1B—C4B—C5B	172.42 (14)
C3A—C4A—C5A—C6A	−104.9 (5)	C20B—C3B—C4B—N1B	−172.76 (15)
N1A—C4A—C5A—C6AA	−31.8 (2)	C2B—C3B—C4B—N1B	−43.40 (16)
C3A—C4A—C5A—C6AA	−150.0 (2)	C20B—C3B—C4B—C5B	77.4 (2)
C6A—C5A—C6AA—C7A	−66.6 (5)	C2B—C3B—C4B—C5B	−153.27 (18)
C4A—C5A—C6AA—C7A	40.2 (3)	N1B—C4B—C5B—C6B	32.1 (2)
C4A—C5A—C6A—C7A	−29.4 (6)	C3B—C4B—C5B—C6B	141.6 (2)
C6AA—C5A—C6A—C7A	56.6 (5)	C4B—C5B—C6B—C7B	−11.0 (3)
C1A—N1A—C7A—C6AA	143.2 (2)	C4B—N1B—C7B—C6B	37.4 (2)
C4A—N1A—C7A—C6AA	13.9 (2)	C1B—N1B—C7B—C6B	173.35 (19)
C1A—N1A—C7A—C6A	101.0 (4)	C5B—C6B—C7B—N1B	−14.6 (3)
C4A—N1A—C7A—C6A	−28.3 (4)	C9B—N2B—C8B—O1B	170.51 (18)
C5A—C6AA—C7A—N1A	−33.8 (3)	C9B—N2B—C8B—C1B	−2.9 (2)
C5A—C6AA—C7A—C6A	57.0 (4)	N1B—C1B—C8B—O1B	−53.8 (2)
C5A—C6A—C7A—N1A	35.3 (6)	C14B—C1B—C8B—O1B	−171.20 (18)
C5A—C6A—C7A—C6AA	−64.2 (5)	C2B—C1B—C8B—O1B	61.0 (2)
C9A—N2A—C8A—O1A	−163.96 (16)	N1B—C1B—C8B—N2B	119.57 (16)
C9A—N2A—C8A—C1A	9.70 (19)	C14B—C1B—C8B—N2B	2.15 (18)
N1A—C1A—C8A—O1A	37.5 (2)	C2B—C1B—C8B—N2B	−125.61 (15)
C14A—C1A—C8A—O1A	163.20 (16)	C8B—N2B—C9B—C10B	−173.85 (19)
C2A—C1A—C8A—O1A	−75.6 (2)	C8B—N2B—C9B—C14B	2.4 (2)
N1A—C1A—C8A—N2A	−136.14 (14)	C14B—C9B—C10B—C11B	−1.5 (3)
C14A—C1A—C8A—N2A	−10.48 (16)	N2B—C9B—C10B—C11B	174.38 (19)
C2A—C1A—C8A—N2A	110.73 (15)	C9B—C10B—C11B—C12B	0.5 (3)
C8A—N2A—C9A—C10A	174.03 (18)	C10B—C11B—C12B—C13B	0.3 (3)
C8A—N2A—C9A—C14A	−4.5 (2)	C11B—C12B—C13B—C14B	−0.1 (3)
C14A—C9A—C10A—C11A	0.9 (3)	C12B—C13B—C14B—C9B	−0.9 (3)
N2A—C9A—C10A—C11A	−177.47 (18)	C12B—C13B—C14B—C1B	−173.48 (18)
C9A—C10A—C11A—C12A	−0.6 (3)	C10B—C9B—C14B—C13B	1.7 (3)
C10A—C11A—C12A—C13A	−0.3 (3)	N2B—C9B—C14B—C13B	−174.85 (17)
C11A—C12A—C13A—C14A	0.9 (3)	C10B—C9B—C14B—C1B	175.73 (17)
C12A—C13A—C14A—C9A	−0.7 (3)	N2B—C9B—C14B—C1B	−0.9 (2)
C12A—C13A—C14A—C1A	−179.14 (17)	N1B—C1B—C14B—C13B	52.7 (2)
C10A—C9A—C14A—C13A	−0.3 (3)	C8B—C1B—C14B—C13B	172.39 (19)
N2A—C9A—C14A—C13A	178.37 (15)	C2B—C1B—C14B—C13B	−63.0 (3)
C10A—C9A—C14A—C1A	178.56 (16)	N1B—C1B—C14B—C9B	−120.42 (16)
N2A—C9A—C14A—C1A	−2.82 (19)	C8B—C1B—C14B—C9B	−0.75 (18)
N1A—C1A—C14A—C13A	−57.0 (2)	C2B—C1B—C14B—C9B	123.81 (16)
C8A—C1A—C14A—C13A	−173.61 (18)	C18B—C2B—C15B—O2B	−136.52 (19)
C2A—C1A—C14A—C13A	64.0 (2)	C3B—C2B—C15B—O2B	−13.1 (3)
N1A—C1A—C14A—C9A	124.40 (15)	C1B—C2B—C15B—O2B	101.1 (2)
C8A—C1A—C14A—C9A	7.79 (17)	C18B—C2B—C15B—C16B	43.8 (2)
C2A—C1A—C14A—C9A	−114.62 (15)	C3B—C2B—C15B—C16B	167.16 (16)
C18A—C2A—C15A—O2A	136.59 (16)	C1B—C2B—C15B—C16B	−78.6 (2)
C3A—C2A—C15A—O2A	11.3 (2)	O2B—C15B—C16B—C17B	139.0 (2)
C1A—C2A—C15A—O2A	−101.35 (18)	C2B—C15B—C16B—C17B	−41.3 (2)
C18A—C2A—C15A—C16A	−41.64 (19)	C18B—N3B—C17B—C16B	−63.3 (2)
C3A—C2A—C15A—C16A	−166.96 (14)	C19B—N3B—C17B—C16B	172.04 (16)
C1A—C2A—C15A—C16A	80.42 (18)	C15B—C16B—C17B—N3B	49.1 (2)
O2A—C15A—C16A—C17A	−136.01 (18)	C17B—N3B—C18B—C2B	69.51 (19)
C2A—C15A—C16A—C17A	42.2 (2)	C19B—N3B—C18B—C2B	−165.34 (16)
C18A—N3A—C17A—C16A	65.47 (19)	C15B—C2B—C18B—N3B	−56.88 (18)
C19A—N3A—C17A—C16A	−166.27 (16)	C3B—C2B—C18B—N3B	179.24 (14)
C15A—C16A—C17A—N3A	−51.2 (2)	C1B—C2B—C18B—N3B	63.43 (19)
C17A—N3A—C18A—C2A	−67.97 (18)	C4B—C3B—C20B—C25B	19.5 (3)
C19A—N3A—C18A—C2A	163.22 (15)	C2B—C3B—C20B—C25B	−102.2 (2)
C15A—C2A—C18A—N3A	52.32 (17)	C4B—C3B—C20B—C21B	−157.21 (18)
C3A—C2A—C18A—N3A	175.61 (13)	C2B—C3B—C20B—C21B	81.1 (2)
C1A—C2A—C18A—N3A	−70.49 (16)	C25B—C20B—C21B—C22B	0.4 (3)
C4A—C3A—C20A—C25A	−36.6 (2)	C3B—C20B—C21B—C22B	177.25 (19)
C2A—C3A—C20A—C25A	86.09 (19)	C20B—C21B—C22B—C23B	−0.5 (3)
C4A—C3A—C20A—C21A	141.23 (16)	C21B—C22B—C23B—C24B	0.2 (3)
C2A—C3A—C20A—C21A	−96.12 (18)	C21B—C22B—C23B—C26B	−179.6 (2)
C25A—C20A—C21A—C22A	−0.2 (3)	C22B—C23B—C24B—C25B	0.2 (3)
C3A—C20A—C21A—C22A	−178.11 (17)	C26B—C23B—C24B—C25B	179.9 (2)
C20A—C21A—C22A—C23A	0.4 (3)	C21B—C20B—C25B—C24B	0.0 (3)
C21A—C22A—C23A—C24A	−0.2 (3)	C3B—C20B—C25B—C24B	−176.80 (18)
C21A—C22A—C23A—C26A	178.3 (2)	C23B—C24B—C25B—C20B	−0.3 (3)

### Hydrogen-bond geometry (Å, º)

Cg1 and Cg2 are the centroids of the C20A–C25A and C20B–C25B rings, respectively.

**Table:**

*D*—H···*A*	*D*—H	H···*A*	*D*···*A*	*D*—H···*A*
N2*A*—H2*A*···O1*A*^i^	0.86	1.99	2.8331 (17)	167
N2*B*—H2*B*···O1*A*^ii^	0.86	2.48	3.0829 (18)	128
N2*B*—H2*B*···N1*A*^ii^	0.86	2.24	3.0461 (18)	156
C4*A*—H4*A*···O1*B*^iii^	0.98	2.52	3.4598 (19)	160
C16*B*—H16*C*···O2*A*	0.97	2.47	3.431 (2)	171
C5*A*—H52*A*···O2*B*	0.97	2.58	3.404 (2)	143
C6*A*—H63*A*···*Cg*1^iv^	0.97	2.97	3.819 (9)	147
C6*B*—H62*B*···*Cg*2^iv^	0.97	2.93	3.827 (3)	155

Symmetry codes: (i) −*x*+2, −*y*+2, −*z*+1; (ii) *x*, *y*−1, *z*; (iii) *x*, *y*+1, *z*; (iv) *x*+1, *y*, *z*.
